# Epigenetic Changes in the Pathogenesis of Rheumatoid Arthritis

**DOI:** 10.3389/fgene.2019.00570

**Published:** 2019-06-14

**Authors:** Marina V. Nemtsova, Dmitry V. Zaletaev, Irina V. Bure, Dmitry S. Mikhaylenko, Ekaterina B. Kuznetsova, Ekaterina A. Alekseeva, Marina I. Beloukhova, Andrei A. Deviatkin, Alexander N. Lukashev, Andrey A. Zamyatnin

**Affiliations:** ^1^Institute of Molecular Medicine, I.M. Sechenov First Moscow State Medical University (Sechenov University), Moscow, Russia; ^2^Laboratory of Epigenetics, Research Centre for Medical Genetics, Moscow, Russia; ^3^Martsinovsky Institute of Medical Parasitology, Tropical and Vector Borne Diseases, I.M. Sechenov First Moscow State Medical University (Sechenov University), Moscow, Russia; ^4^A.N. Belozersky Institute of Physico-Chemical Biology, Lomonosov Moscow State University, Moscow, Russia

**Keywords:** rheumatoid arthritis, epigenetics, DNA methylation, miRNA, histone modifications, circRNA

## Abstract

Rheumatoid arthritis (RA) is a systemic autoimmune disease that affects about 1% of the world’s population. The etiology of RA remains unknown. It is considered to occur in the presence of genetic and environmental factors. An increasing body of evidence pinpoints that epigenetic modifications play an important role in the regulation of RA pathogenesis. Epigenetics causes heritable phenotype changes that are not determined by changes in the DNA sequence. The major epigenetic mechanisms include DNA methylation, histone proteins modifications and changes in gene expression caused by microRNAs and other non-coding RNAs. These modifications are reversible and could be modulated by diet, drugs, and other environmental factors. Specific changes in DNA methylation, histone modifications and abnormal expression of non-coding RNAs associated with RA have already been identified. This review focuses on the role of these multiple epigenetic factors in the pathogenesis and progression of the disease, not only in synovial fibroblasts, immune cells, but also in the peripheral blood of patients with RA, which clearly shows their high diagnostic potential and promising targets for therapy in the future.

## Introduction

Rheumatoid arthritis (RA) is a chronic auto-inflammatory disease of connective tissue with progressive joint damage and systemic disorders that affects around 1% of the world’s population (Cribbs A. et al., 2015). RA can cause various symptoms, clinical forms and prognoses. The incidence of RA begins to increase at the age of 25 years; at the age of 55, the incidence of RA is reaching a plateau (Gabriel, 2001). For example, RA is more than six times greater in 60- to 64-year-old women compared to 18- to 29-year-old women (Melorose et al., 2015). The prevalence of RA varies in different ethnic groups. For example, the incidence of RA among American Indians is 7%, while for some other nations it is 0.2–0.4% (Ferucci et al., 2005). As in most other autoimmune diseases, RA is more common among women than among men in a ratio of 2–3 to 1 (van Vollenhoven, 2009). Based on this fact, there are assumptions that estrogens are actively involved in the pathogenesis of the disease (Wluka et al., 2000).

The etiology of RA remains unknown. It is considered to occur in the presence of genetic predispositions and provoking environmental factors. The heritability of RA has been shown from twin studies to be 60% (Yarwood et al., 2016). Earlier genealogical studies and modern molecular-genetic investigations confirm the involvement of genetic factors in RA development. Accumulation of the disease cases was revealed within families along with an increased risk of RA among first-degree relatives of the patients (Sparks and Costenbader, 2014).

The pathological process in RA represents an autoimmune inflammation of the synovial membrane of joints with synovial cells proliferation and pannus formation. This tumor-like aggressive granulation tissue promotes articular cartilage erosion and bones destruction. Synovial tissue dysfunction allows macrophages, fibroblasts and activated lymphocytes to penetrate into it. T-lymphocytes produce a variety of proinflammatory cytokines, predominantly belonging to tumor necrosis factor (TNF) and interleukin (IL) superfamilies as well as growth factors (Firenstein et al., 2013). B-lymphocytes are involved in the production of autoantibodies such as rheumatoid factor (RF) and antibodies against cyclic citrullinated peptide (anti-CCP). Differencies in expression of anti-CCP and RF, rate of disease manifestation and variability of response to therapy cause heterogeneity of RA patients indicating different pathophysiological mechanisms implication in the disease development and progression.

Genetic heterogeneity does not explain all the features of RA (Viatte et al., 2013). Thus, investigation of epigenetic factors and mechanisms associated with the progression of the disease and response to treatment is increasingly important. Investigation of the epigenetic landscape can provide novel therapeutic targets (Glant et al., 2014).

Different levels of DNA organization and chromatin packing in the eukaryotic cell’s nucleus are points of application for the epigenetic regulation. Epigenetic mechanisms regulate chromatin structure and create clear patterns of gene expression during cell differentiation.

The chromatin structure regulates gene transcription by altering DNA regulatory regions (such as promoters and enhancers) availability for transcription factors (TF). An open chromatin structure, euchromatin, enables DNA-binding proteins and TF to interact with regulatory DNA sequences, leading to active gene transcription. Conversely, heterochromatin is a closed condensed chromatin state, where DNA is tightly bound to protein complexes forming a superspiralized structure. It prevents TF interaction with regulatory sequences, thus inactivating gene expression and leading to its silencing. Transcription factors, non-coding RNAs (ncRNAs), DNA methylation, histone modification and microRNAs (miRNAs) affect gene transcription without changing the DNA sequence itself (reviewed by Golbabapour et al., 2011). Specific epigenetic landscape of chromatin determines a differential gene expression and regulates various cellular processes in physiological and pathological conditions.

Epigenetic changes in RA have been studied both in mononuclear cells of peripheral blood and in different types of immune cells such as monocytes, T-cells and B-cells (Ospelt, 2016). At the same time, the epigenetic modifications in the rheumatoid arthritis synovial fibroblasts (RASFs) are of particular interest because of their aggressive phenotype, which is stable for several passages in cell culture (Hardy et al., 2013). RASFs are clue cells of joint damage and inflammation development due to pro-inflammatory and catabolic molecules synthesis, promoting abnormal proliferation and invasiveness. Implantation of RASFs together with normal human cartilage to immunodeficient mice revealed cell attachment and cartilage destruction without any proinflammatory stimuli. Such behavior was not observed in osteoarthritis (OA) synovial fibroblasts and is presumably related to epigenetic changes in these cells due to the specific pathology only (Lefèvre et al., 2009).

## Aberrant DNA Methylation in Immune Cells and Peripheral Blood Cells in RA

DNA methylation is a biochemical process of methyl group binding with the cytosine ring carbon at position 5 to form 5-methylcytosine (5-mC). In mammals, DNA methylation occurs preferentially in CpG dinucleotides located throughout the whole gene either as single dinucleotide or concentrated into CpG-islands in vicinity of gene promoters. Hypermethylation of the promoters is an indicator of dense heterochromatin conformation, which blocks the binding of TF to DNA and leads to inactivation of gene transcription. The low-level methylation of promoters (hypomethylation) is associated with open chromatin conformation and active transcription of the gene (Eden and Cedar, 1994). DNA methylation is a reversible process, which could therefore be considered as a therapeutic target.

Recent studies confirmed a global DNA hypomethylation in T-cells and monocytes of RA patients compared to healthy individuals (de Andres et al., 2015). Genome-wide analysis of DNA methylation by microarrays revealed its alterations in B-cells on the early stages of RA in patients who have not yet received treatment compared to healthy donors (Glossop et al., 2016).

Cribbs et al. (2014) analyzed an aberrant function of regulatory T cells (Treg) in RA patients and found a specific region in the promoter of the *CTLA-4* (-658 CpG), which was hypermethylated in comparison with healthy controls. DNA hypermethylation prevents binding of the nuclear factor of activated T cells (NF-AT) with cytoplasmic one, called NF-ATc2, which leads to decrease of *CTLA-4* expression. As a consequence, Treg cells were unable to induce expression and activation of the tryptophan-degrading enzyme indoleamine 2,3-dioxygenase (IDO), which in turn resulted in a failure to activate the immunomodulatory kynurenine pathway (Cribbs et al., 2014). Furthermore, treatment with methotrexate induced DNA hypomethylation of *FoxP3* locus in Treg. This results in the gene upregulation with consequent increase of CTLA-4 concentration and normalization of Treg function in RA. These studies clearly illustrate how aberrant DNA methylation can affect cell functions and how epigenetic mechanisms can be used in therapy (Cribbs A.P. et al., 2015). To determine differentially methylated regions as potential epigenetic risk factors and markers of RA predispositions, Liu et al. (2013) performed epigenome-wide association study. Using Illumina 450k microarrays they examined more than 485,000 CpG sites in peripheral blood of 354 RA patients and 337 healthy donors. As a result, 10 differentially methylated CpG sites were identified. All of them are localized on 6p12.1 and form two separate clusters within the locus also containing the genes of the major histocompatibility complex (MHC) that is known as the risk locus of RA (Raychaudhuri et al., 2012). This confirms the role of DNA methylation as an additional mechanism determining susceptibility to RA. Importantly, the heterogeneity of cell population isolated from a whole blood may cause diverse methylation profile. Thus, this factor should be taken into account in bioinformatic analysis to reduce possible biases.

Some of these results were confirmed by other studies. Aberrant DNA methylation was detected in peripheral blood mononuclear cells (PBMCs) of RA patients. For example, van Steenbergen et al. (2014) demonstrated that cg23325723 site was significantly associated with RA (*p* = 0.026) in PBMCs. Four other CpG sites (cg16609995, cg19555708, cg19321684, and cg25949002) demonstrated similar different methylation in PBMCs comparing to control samples, which was not, however, statistically significant.

Other studies have shown abnormal methylation of one cytosine in the *IL-6* promoter in RA PBMCs associated with reduction of its transcription (Nile et al., 2008). At the same time the loss of cytosine methylation in the *IL-10* promoter correlates with higher expression of IL-10 in such cells (Chen et al., 2011).

*LRPAP1* gene is expressed in PBMCs and encodes the chaperone of low density lipoprotein receptor-related protein 1, that affects the activity of transforming growth factor beta (TGF-β) (Kolker et al., 2012). It was found that 4 CpG-dinucleotides in exon 7 of *LRPAP1* were hypermethylated in patients who demonstrated no response to the therapy by TNF inhibitors (etanercept) compared to responders. The locus of cg04857395 overlaps structures involved in alternative splicing: the region associated with trimethylation of histone H3 at lysine 36 (H3K36me3) and the binding site of CCCTC-binding factor, which is a methyl-sensitive transcriptional repressor (Lev Maor et al., 2015).

An important point to consider in epigenetic studies of PBMCs is the effect of cell heterogeneity. If the experimental data are not normalized according to the proportion of the cells of different types in the fraction of PBMCs, the differentially methylated regions (DMRs) in certain cell types could be missed.

DNA methylation in peripheral blood mononuclear cells was recently described by Zhu et al. (2019). DNA methylation profiling and gene expression profiling were measured in patients with RA and in healthy controls. Differentially methylated sites and genes identified an interferon inducible gene interaction network. The significance of PARP9 gene methylation and its associated change in the expression in the pathogenesis of RA was demonstrated. In addition, its ability to positively regulate IL2, which stimulates various cells of the immune response, has been revealed (Zhu et al., 2019).

Epigenetic regulation of immune cells can be crucial for the development and maintenance of autoimmune diseases, such as RA. Julià et al. (2017) investigated the methylation patterns of B lymphocytes in patients with RA and systemic lupus erythematosus. Differentially methylated in patients and in the control group CpG sites were located in the CD1C, TNFSF10, PARVG, NID1, DHRS12, ITPK1, ACSF3, and TNFRSF13C genes and two intergenic regions (10p12.31). Differential methylation of these genes was also reproduced in the cohort of patients with SLE. This indicates similar patterns of epigenetic changes in B-lymphocytes in these two autoimmune diseases (Julià et al., 2017).

## Aberrations of DNA Methylation in Synovial Fibroblasts in RA

Rheumatoid arthritis synovial fibroblasts (RASFs) have a unique, non-random methylation pattern - methylome, which is specifically reorganized during the disease progression and varies depending on the joint localization. A precise mechanism of the methylome changing remains still unclear, but the overall pattern of differential methylation corresponds to the aggressive phenotype acquisition in SF results in the development of the disease. Earlier studies of epigenetic changes in RASFs demonstrated an abnormal expression of retroviral sequences LINE-1, associated with loss of silencing of these mobile elements as a result of hypomethylation (Neidhart et al., 2000). Global DNA hypomethylation is observed in many hyperproliferating tissues and is associated with a relative lack of methyl groups’ donor *S*-adenosylmethionine (SAM). SAM is required to restore the DNA methylation after cell division, as well as for polyamines recycling. Increased cell proliferation leads to increased polyamines processing, competing with DNA methylation (Brooks, 2012). Interestingly, the key enzymes that are involved in the polyamine synthesis are encoded in the X chromosome and an elevated level of polyamines is found in many autoimmune diseases including RA (Furumitsu et al., 1993). Thus, high level of polyamines is associated not only with DNA hypomethylation, but also with an increased risk of RA development in women.

Nakano et al. (2013a) have shown that DNA hypomethylation increases expression of numerous genes: growth factors/ receptors, extracellular matrix proteins, adhesion molecules, and matrix degrading enzymes, etc. Expression of the DNA methyltransferase-1 (DNMT1) is reduced on protein level in RASFs comparing to the osteoarthritis synovial fibroblasts (OASF), particularly when stimulated with cytokines or growth factors. However, DNMT1A transcripts levels are similar in both of these cell types. Additionally, transcription of DNMT1 could be reduced by stimulation of IL-1 (Nakano et al., 2013a).

A number of differentially methylated loci were reported in RASFs. For example, hypomethylation of *CXCL12* (chemokine C-X-C motif ligand 12) promoter is associated with the gene’s upregulation and accumulation of the protein in the joints of RA patients that contributes to chronic inflammation. TBX5 regulates expression of proinflammatory cytokines and chemokines in SF including CXCL12 chemokine, which is the downstream effector of the same pathway. Hypomethylation of *TBX5* (T-box transcription factor 5) increases its own expression as well as *CXCL12* expression in RASFs. Treatment of SF cell culture with 5-aza-2′-deoxycitidine (5-aza-dC) to achieve DNA demethylation induces hypomethylation of promoter region and subsequent re-activation of CXCL12 expression (Karouzakis et al., 2014).

Comprehensive analysis of DNA methylation in RASFs by Nakano et al. (2013b) identified 1 859 differently methylated loci. Some of the hypomethylated loci that were critically important for RA pathogenesis were located in the genes *CHI3L1*, *CASP1*, *STAT3*, *MAP3K5*, *MEFV*, and *WISP3*. Conversely, genes *TGFBR2* and *FOXO1* were hypermethylated. As shown by analyzing regulatory pathways, the aberrantly methylated genes were involved in cell migration, adhesion, transendothelial penetration and interactions in the extracellular matrix (Nakano et al., 2013b).

Analyzed patterns of DNA methylation suggest its aberrations do not occur randomly, but are specifically related to regulatory pathways involved in RA pathogenesis. Interestingly, RASFs DNA methylation patterns are altering during disease development and progression from early to chronic stage (Whitaker et al., 2013).

A recent study reported genome differently methylated sites that are localized in CpG-islands regions in promoters of RA patients with different clinical symptoms and age of manifestation (Karouzakis et al., 2018). Specifically methylated CpG-islands were found on every stage of the disease. Significant hypermethylation of CpG-islands was revealed in the promoters of peptidase M20 containing domain-1 gene (*PM20D1*), *SHROOM1* and engrailed-1 homeobox protein (*EN1*) at very early RASFs compared to normal SFs. *SHROOM1* gene is involved in the development of nervous tissue and the rearrangement of microtubules during cell division. The chondrocytes of knee joints in early RA and transient arthritis patients significantly differ in *SHROOM1* methylation making it a valuable biomarker for early diagnostics of the disease (Bonin et al., 2016).

Another set of genes with hypermethylated promoters has been identified in patients with chronic disease: microfibrillar-associated protein 2 (*MFAP2*), discoidin domain receptor (*DDR1*) tyrosine kinase and the major histocompatibility complex *HLA-C*. Several identified CpG-islands were specifically hypermethylated in the SFs of very early and/or chronic RA. The MFAP2 binds TGFβ and the members of bone morphogenetic protein (BMP) family, thus regulates release and activation of these factors involved in the development of arthritis (Weinbaum et al., 2008). Tyrosine kinase DDR1 binds collagens and can regulate various cellular processes such as cell migration, invasion, and proliferation (Juskaite et al., 2017). This indicates promising targets for further functional experiments that could explain the phenotypic changes in RASFs and their invasive behavior.

To identify novel therapeutic targets for RA treatment, the analysis of the methylome was recently suggested along with other data on RASFs. Whitaker et al. (2015) have combined findings from genome-wide association studies and analysis of differential gene expression and DNA methylation analysis in RASFs and OASFs. As a result, a number of genes were chosen as prominent candidates for further investigation relevant for RA pathogenesis: *ELMO1*, *LBH*, and *PTPN11*, which are directly involved in the pathogenesis of RA and may be used as therapeutic targets.

*ELMO1* encodes a protein involved in cytoskeleton reorganization, which is crucial for phagocytosis of apoptotic cells and cell motility. *ELMO1* promoter is hypermethylated in RASFs, and its knockdown suppresses the RASFs migration and invasion by reducing the activation of RAC1 GTPase (Whitaker et al., 2015). These results demonstrate how the integration of datasets from genome-wide methylation and gene expression analyses allows identifying proteins with previously unknown critical role in RA development. Such a complex “omics” approach can be extended from studying only promoter regions of the genes to enhancers, silencers and other regulatory sequences with almost unknown effect of methylation. The application of such approaches led to discovery of novel differentially methylated loci in the RASFs.

Previously it was thought a protein encoded by *LBH* contributes only in embryonic development. However, *LBH* promotor was found to be hypomethylated in RASFs as well as its enhancer. The gene knockdown affected the transcriptome including pathways that control cell growth and proliferation in RASFs (Ekwall et al., 2015). Interestingly, its enhancer domain contains single nucleotide polymorphism (SNP) rs906868 associated with RA. The combination of the SNP genotype and methylation affects the activity of enhancer and, consequently, expression of *LBH* (Hammaker et al., 2016).

*PTPN11* encodes the tyrosine phosphatase SHP2 and is upregulated in RASFs (Stanford et al., 2013). Analysis of the *PTPN11* enhancer in RASFs revealed hypermethylation, which increased the sensitivity of cells to glucocorticoids and their aggressiveness. This not only explained the mechanism of action of PTPN11 in RASFs but also demonstrated SHP2 as a potential therapeutic target in RA. The results were confirmed on mouse models of arthritis (Whitaker et al., 2016).

Summing up, analysis of the methylome in RASFs contributes to understanding of RA pathogenesis. Not only local cytokine environment but other factors can potentially affect DNA methylation pattern and participate in establishing a stable phenotype of RASFs. Identification of these environmental factors could shed light on the predisposition to RA development and progression. In addition, studies of RASFs used the novel “omics” technologies – include not only methylome but also the other types of epigenetic markers – will help discover novel molecular factors in the RA pathogenesis and determine potential therapeutic targets. Considering the role of differentially methylated genes from the pathway perspective, several cascades were reported to be usually disturbed in very early RASFs and chronic RASFs in comparison with normal SFs but exhibit no changes in the SFs in transient arthritis. These regulatory pathways include cadherin, integrin, WNT signaling of cell adhesion, components of the actin cytoskeleton and antigen presentation.

## Histone Modifications in Immune Cells in RA

Histone modifications are important epigenetic marks that affect gene expression and determine phenotype of cells. Histone proteins are bound to DNA and regulate the accessibility of gene promoters for transcription factors. The basic functional unit of chromatin is the nucleosome. It contains 147 base pairs of DNA, which are wrapped around a histone octamer that consists of two copies each of histones H2A, H2B, H3, and H4. The epigenetic landscape of histones can be modified by numerous mechanisms including acetylation, methylation, citrullination, phosphorylation, ubiquitinylation, and sumoylation (Tessarz and Kouzarides, 2014). Some histone marks are associated with an open chromatin structure. This makes chromatin accessible to transcription factors and can significantly increase gene expression. These include histones lysine residues acetylation (H3K9, H3K14, H4K5, and H4K16) and methylation (H2BK5, H3K4, H3K36, and H3K79); phosphorylation of histone H3 threonine 3 (H3T3) and serines (H3S10 and H3S28), and as well as histone 4 serine 1 (H4S1) and H2BK120 ubiquitinylation. On the other hand, repressive histone marks that correlate with heterochromatin state and gene repression include methylation of H3K9, H3K27, and H4K20; ubiquitination of H2AK119; and sumoylation of H2AK126, H2BK6, and H2BK7 (Araki and Mimura, 2016). Most studies have focused on the acetylation and methylation of histones, although citrullinated histones in RASFs has also been reported (Wang et al., 2016).

Different types of histone modifications are colocalized throughout the genome in order to stabilize active or repressed chromatin states. This complicates analysis of RA-specific alterations of histone marks. The expression of histone-modifying enzymes (e.g., histone deacetylases and HDAC) could be studied instead. The interest to the expression of HDAC in RA arose after reports on its inhibitors, which were considered as novel and promising therapeutic strategy of inflammatory diseases. One of the studies revealed activation of HDAC in PBMCs of RA patients compared to healthy controls (Toussirot et al., 2013). Controversial data were also obtained for HDAC activity in the synovial tissues of RA patients. Presumably, HDAC activity depends strongly to the disease progression and therapy. This may explain discrepancies in the measurements among different cohorts of patients whereas the changes that were identified in larger and clearly defined groups got lost.

## Histone Modifications in RASFs

Similarly as in immune cells, direct studies of histone modifications in synovial fibroblasts are quite rare. Huber et al. (2007) demonstrated an overall increase of acetylation associated with reduced HDAC activity as a result of decreased *HDAC1* and *HDAC2* gene expression in the synovial tissue of RA patients. Suppression of HDAC1 and HDAC2 suggests the balance between histone acetylases (HAT) and HDAC activity shifts toward hyperacetylation in RA synovial tissues. However, another study demonstrated that HDAC activity and *HDAC1* expression were upregulated in RA synovial tissue (Kawabata et al., 2010). Not all members of the HDAC family have pro-inflammatory effect. For example, HDAC5 demonstrates anti-inflammatory functions in SFs. That indicates the applicability of specific rather than general HDAC inhibitors for the RA treatment (Angiolilli et al., 2016). Recently, suppression of HDAC3 expression was found to be as effective in suppressing pro-inflammatory factors in the RASFs as general inhibition of HDAC, which makes HDAC3 a promising candidate for targeted therapy (Angiolilli et al., 2017).

### Abnormal Modifications of Histones Are Involved in the Activation of RASFs

H3K27 specific histone methyltransferase (HMT) – the enhancer of zeste homolog 2 (EZH2) – is highly expressed in RASFs as a result of TNFα induction of nuclear factor kappa B (NF-KB) and mitogen-activated protein kinase (MAPK) pathways. The expression of the secreted frizzled-related protein 1 (SFRP1) – a EZH2 target gene – is increased under the active histone marks (H3K4me3 and H3K27me3) in its promoter that leads to the Wnt (wingless-type MMTV integration site signaling)-pathway inhibition in RASFs (Trenkmann et al., 2011).

Transcription factor T-box transcription factor 5 (TBX5) is overexpressed in RASFs and active histone marks – including H4K4me3 and histone acetylation – are widely represented in its promoter. The overexpression of TBX5 affects expression of 790 genes including *IL-8*, chemokine C-X-C motif ligand 12 (*CXCL12*) and chemokine C-C motif ligand 20 (*CCL20*) confirming its role as an inductor and regulator of chemokines important in RA development (Karouzakis et al., 2014).

Ai et al. (2018) performed the comprehensive study, describing histone modification, open chromatin, RNA expression, and genome-wide DNA methylation in synovial fibroblasts in RA patients. To determine complex multidimensional interactions in the epigenetic regulation of RA, an integrative analysis was performed using a new method for detecting genomic regions with similar profiles. In addition to the known pathological pathways that are activated in RA, the authors found novel pathway activated in RA that was previously known to be associated with Huntington’s disease (Ai et al., 2018).

In the other paper (Webster et al., 2018) epigenetic changes in RASF in 79 pairs of discordant on RA monozygotic twins were revealed. An epigenetic signature has been shown to indicate the association of stress response pathways and RA pathogenesis. It is noteworthy that potential epigenetic disruption of multiple RUNX3 transcription factor binding sites was proposed to be associated with disease development.

### Histones Modifications Affect Matrix Metalloproteinase Genes Regulation

Araki et al. (2015) reported significantly higher levels of the activating trimethylation mark H3K4me3 in promoters of *MMP-1*, *MMP-3*, *MMP-9*, and *MMP-13* along with reduced of the repressive modification H3K27me3 in promoters of *MMP-1* and *MMP-9* in RASFs. Furthermore, the elevated level of histone H4 acetylation was associated with upregulation of *MMP-1* (Maciejewska-Rodrigues et al., 2010).

Tryptophan-aspartate (WD) repeat-containing protein 5 (*WDR5*) is a major subunit of the proteins bound with SET1 (COMPASS) or COMPASS-like complexes that catalyze H3K4 methylation. *WDR5* knockdown reduces the level of H3K4me3 marks as well as the abundance of MMP-1, MMP-3, MMP-9, and MMP-13 in RASFs. IL-6 and soluble IL-6 receptor α (sIL-6Rα) induce the expression of *MMP-1*, *MMP-3*, and *MMP-13* but not of *MMP-9*: It has been shown that IL-6-induced signal transducer and activator of transcription 3 (STAT3) binds to *MMP-1*, *MMP-3*, and *MMP-13* promoters but not with that of *MMP-9*. High expression of IL-6 was associated with high level of histone H3 acetylation (H3ac) of the IL-6 promoter in RASFs (Wada et al., 2014).

## MicroRNAs as an Epigenetic Factor Associated with the Development of RA

MicroRNAs (miRNAs) are small non-coding RNAs of 17–25 nt that regulate gene expression by either repressing the translation or causing degradation of multiple target mRNAs (Fabian et al., 2010). miRNAs play an important role in many biological processes including the development of the immune system and the subsequent regulation of immunity both innate and acquired (Chen et al., 2016). Currently more than 100 miRNAs have been identified that could potentially affect the molecular pathways in immune cells development and their functions regulation (Baulina et al., 2016).

## Aberrations of MicroRNA Expression in RA

Aberrant miRNA regulation occurs in various cells and tissues in RA (Tavasolian et al., 2018). The role of miRNA in the inflammatory process includes both control of cytokine production and protection of cartilage tissue by regulating catabolic activity, proliferation and resistance to apoptosis.

The increased production of IL-17 by T helper cells (Th17) in the synovial fluid may suppress miRNA-23b and so enhance expression of (TGF)-β-activated kinase 1/MAP3K7-binding protein and Iκβ kinase α contributing to inflammation. miRNA-21 promotes the differentiation of Th2 and Treg cells and is also associated with the regulation of Treg apoptosis (Salehi et al., 2015). Pro-inflammatory phenotype of Treg is determined by an aberrant miRNA-146a expression; thus its reduction in RA patients inversely correlated with disease activity and expression of its direct target gene *STAT1* (Zhou et al., 2015).

A number of studies have described miRNAs that modulate inflammatory or catabolic functions of RASFs thereby contributing to the development of the aggressive phenotype in RA (Vicente et al., 2016).

miRNA-146 and miRNA-155 were first described as abnormally expressed in synovial tissue, RASFs and synovial fluid of patients with RA and still remain the best characterized candidates. miRNA-155 is upregulated in RASFs, where – in addition to the pro-inflammatory activity – it regulates destructive processes due to the matrix metalloproteinases MMP-1 and MMP-3 expression repressing (Long et al., 2013). Similarly, miRNA-146a express increasingly in RASFs, despite a known role as a negative regulator of inflammation in immune cells (Vicente et al., 2016).

Increased expression in RASFs has also been determined for a number of other transcripts: miRNA-203 (Stanczyk et al., 2011), miRNA-221 (Yang and Yang, 2015), miRNA-663 (Miao et al., 2015a), miRNA-222 and miRNA-323-3p (Pandis et al., 2012). Besides their role in immune processes, these microRNAs are involved in oncogenesis by regulating cell invasiveness and migration in different types of tumors.

Earlier reports show miRNA-203 overexpression associated with hypomethylation of MMP1 and IL-6 gene promoters induces these proteins production. Regulation by DNA methylation was also observed in mir-203. Interestingly, this miRNA high-level expression in RASFs is observed with its own promoter hypomethylation (Stanczyk et al., 2011).

Suppression of miRNA-221 inhibits the production of pro-inflammatory cytokines by fibroblasts, causes cell apoptosis and reduces their migration and invasion of RASFs (Yang and Yang, 2015).

miRNA-663 regulates proliferation of RASFs and production of IL-6 via inhibition of the tumor suppressor *APC* thereby affecting the Wnt signaling pathway (Miao et al., 2015a).

Overexpression of miRNA-124a in RASFs may disrupt the cell cycle and lead to inhibition of cell proliferation through repression of its target genes *CDK-2* and *MCP-1*. Additionally, it was shown that miRNA-124a expression is regulated by methylation of the gene from which it is transcribed. Demethylation of the miRNA-124a gene by 5-aza-dC reduces RASF proliferation and expression of TNF-α (Zhou et al., 2016). Regulation by DNA methylation was also observed in mir-203. Its high expression in RASFs is associated with the promoter hypomethylation (Stanczyk et al., 2011).

The similar effect on RASFs proliferation has miRNA-34a^∗^ (Niederer et al., 2012). It regulates genes of the apoptosis inhibitor XIAP. Furthermore, direct correlation of miRNA-21, miRNA-25, and miRNA-124a from peripheral blood cells with estradiol level in plasma was described in women with RA. The effect of estradiol on miRNA-124a is particularly interesting, as this miRNA has an effect on synovial proliferation (Singh et al., 2013). Decreased expression of both miRNA-124a and miRNA-34a^∗^ as well as the others: miRNA-152, miRNA-375, and miRNA-22 in RASFs contributes to RA development (Vicente et al., 2016).

miRNA-152 and miRNA-375 directly target DNMT1. Thus, the increase of their expression leads to activation of Wnt-signaling pathway (Miao et al., 2015b).

*Cyr61* expression is increased by miRNA-22 suppression, affecting various genes involved in different processes: angiogenesis, inflammation, matrix structure reorganization, IL-6 production with subsequent differentiation of Th17 and synovial hyperplasia. Earlier data show that the reduced expression of miRNA-22 is caused by p53 mutation that is frequent in RASFs (Lin et al., 2014).

The loss of miRNA-10a-5p expression in RASFs upregulates target gene *TBX5* thereby promoting the production of TLR3, MMP-13 and a number of pro-inflammatory cytokines (Hussain et al., 2018). Suppressed expression of miRNA-10a may cause the activation of NF-κB and enhancing the release of pro-inflammatory cytokines TNF-α, IL-1β, IL-6, and IL-8, chemokine MCP-1 and matrix metalloproteinases MMP-1 and MMP-13 (Mu et al., 2016).

Deregulations of expression affect entire miRNA clusters. However, the expression of individual miRNAs of a cluster may be altered differentially. For example, miRNA-18a is upregulated in the miRNA-17-92 cluster, which plays an important role in the regulation of apoptosis in the RASFs while miRNA-19a/b, miRNA-20a and miRNA-30a-3p are downregulated. Such an effect may be caused by the different target genes affection and different signal pathways regulation (Vicente et al., 2016).

The RA associated changes of miRNA expression were also confirmed in other cells of the joint capsule. For example, miRNA-323-3p is involved in the regulation of Wnt and cadherin signaling pathways, and its overexpression in chondrocytes causes degradation of the cartilaginous matrix and promotes bone erosion. Similar effect was demonstrated for miRNA-140, which contributes to reduction of joint destruction via suppression of *ADAMSTS5*. Upregulation of some miRNAs (miRNA-30a, miRNA-204, miRNA-211, miRNA-320, and miRNA-335) is associated with suppression of osteoblast differentiation via RUNX2 regulation (Moran-Moguel et al., 2018).

## Circulating miRNAs as Potential Markers of RA Development

In addition to aberrant miRNAs expression in the joint area, they were also detected in blood: plasma, serum, and various blood cells (Table 1). In one of the first studies of miRNA expression in RA a significant difference between the 26 microRNAs expression patterns was demonstrated in patients compared to healthy donors. Three of those, namely miRNA-24, miRNA-26a, and miRNA-125a-5p have been proposed as a potential diagnostic panel with sensitivity and specificity of 78.4 and 92.3%, respectively (Murata et al., 2013).

**Table 1:** Potential diagnostic and prognostic markers of RA among miRNAs.

Clinical potential	miRNA	Type of sample	Expression	References
**Diagnostic marker**	miRNA-16	Synovial fluid	Increased, comparing to another autoimmune diseases	Mi et al., 2013
	miRNA -223	Synovial fluid	Increased, comparing to another autoimmune diseases	Shibuya et al., 2013
	miRNA-24, miRNA-30a-5p, miRNA-125a-5p	Plasma	Increased in ACPA-positive and ACPA-negative donors before clinical symptoms appear	Murata et al., 2013
	miRNA-22, miRNA-382, miRNA-486-3p	Whole blood	Increased in ACPA-positive donors before clinical symptoms appear	Ouboussad et al., 2017
	miRNA-10a	Whole blood	Decreased in RA patients	Hong et al., 2018
	miRNA-155	Serum	Increased in RA patients	Abdul-Maksoud et al., 2017
	miRNA-210	Serum	Decreased in RA patients	Abdul-Maksoud et al., 2017
	miRNA-26à	Serum/plasma	Increased in RA patients	Murata et al., 2013
**Disease progression**	miRNA-146a, miRNA-16, miRNA-150	PBMCs	Correlates with disease progression	Filkova et al., 2012
	miRNA-16, miRNA-146a, miRNA-155	Whole blood	Lower level on early stages, than in advanced disease	Filková et al., 2014
	miRNA-146a	Synovial fluid, blood	Correlates with disease progression	Chung et al., 2016
	miRNA-16	Plasma	Correlates with disease progression	Murata et al., 2010
**Response to treatment:**			
**Adalimumab**	miR-22	Whole blood	Better response to therapy when decreased	Krintel et al., 2016
	miR-886-3p	Whole blood	Better response to therapy when increased	Krintel et al., 2016
**Rituximab**	miRNA-125b	Serum	Better response to therapy when increased	Duroux-Richard et al., 2014
**Methotrexate**	miR-10a	Whole blood	Better response to therapy when decreased	Hong et al., 2018
**Anti-TNF-α therapy**	miR-5196	Serum	Better response to therapy when increased	Ciechomska et al., 2017
	miR-146a-5p	Serum	Better response to therapy when increased	Bogunia-Kubik et al., 2016
**Anti-TNF/DMARD therapy**	miR-16-5p, miR-23-3p, miR125b-5p, miR-126-3p, miR-146a-5p, miR-223-3p	Serum	Prognostic panel of therapy efficiency	Castro-Villegas et al., 2015
**DMARD therapy and glucocorticoids**	miR-16, miR-223	Serum	Better response to therapy when increased	Filková et al., 2014


Nevertheless, estimation of two other miRNAs expression level is considered more prominent at the moment. miRNA-146a and miRNA-155 are overexpressed both in whole blood and PBMCs of RA patients (Mookherjee and El-Gabalawy, 2013).

Many miRNAs that are aberrantly expressed in synovial tissues in RA are also deregulated in peripheral blood. However, these changes are not always similar. The concentrations of miRNA-16, miRNA-132, and miRNA-223 were significantly lower in the synovial fluid than in plasma of patients with RA, and no correlation between them was found (Murata et al., 2010). Elevated level of miRNA-125b was observed in both serum and synovial tissues of RA patients (Zhang B. et al., 2017).

While miRNA-146a is overexpressed in synovial tissue of RA patients comparing with healthy donors, a reduction of circulating miR-146a was reported in the peripheral blood (Wang et al., 2012). Besides, miRNA-146a from PBMCs does not demonstrate any correlation with disease activity. That is in contrast with the same miRNA quantified in serum. Thus, the miRNAs obtained from different types of samples may have different prognostic value or not have it at all (Ayeldeen et al., 2018).

Changes in the expression of miRNAs may be associated with the RA therapy (Table 1). Specifically, miRNA-146a, miRNA-155, and miRNA-16 levels were decreased in serum at the early RA stages after treatment with disease-modifying antirheumatic drugs (DMARDs) (Filková et al., 2014). In contrast the quantity of miRNA-16-5p, miRNA-23-3p, miRNA125b-5p, miRNA-126-3p, miRNA-146a-5p, and miRNA-223-3p in plasma was significantly elevated after combined anti-TNF-α/DMARD therapy (Castro-Villegas et al., 2015).

### miRNAs Genes Polymorphism Associated With RA

miRNAs not only regulate gene expression in RA but also are themselves subject to regulation by various factors. As in protein-coding genes, polymorphism of miRNA genes or their target genes may be associated with predisposition to RA. The number of studies has reported a significant role of some nucleotide polymorphisms located in the genes of miRNA-146a and miRNA-499 precursors (Ayeldeen et al., 2018). For example, the importance of polymorphism rs3746444 in miRNA-499 was confirmed for the RA development among the patients of Caucasian race. Genotypes TC and CC and allele C at this SNP in miRNA-499 were characterized as independent risk factors of joint erosion in RA patients. The frequency of GG genotype of rs2910164 in miRNA-146a is significantly higher in patients with RA compared to healthy donors (Ayeldeen et al., 2018). Another study that included 200 RA patients and 120 healthy donors has demonstrated the correlation of SNP rs22928323 in miRNA-149 with RA development but no association with further clinical characteristics (Xiao et al., 2015).

### DNA Methylation of miRNA Genes

Similarly to protein-coding genes, DNA methylation is an important mechanism of miRNA regulation. It was demonstrated that miRNA-124a and miRNA-203 are controlled by DNA methylation of respective genes in RASFs. *In vitro* 5-azacitidine treatment leads to DNA demethylation and transcriptional re-activation (Stanczyk et al., 2011; Zhou et al., 2016). de la Rica et al. (2013) provided additional evidence of such epigenetic regulation in the whole genome by profiling of the methylome and analysis of miRNA and mRNA expression in RASFs in parallel. Expression of 11 miRNAs was reduced in RA samples comparing to control osteoarthritis samples and was associated with hypermethylated genes thereof. In contrast, four other miRNAs were upregulated upon hypomethylation of CpG sites in vicinity of their genes (de la Rica et al., 2013).

### Long Non-coding and Circular RNAs in RA

Several long non-coding RNAs (lncRNAs) were deregulated in RA by miRNAs. The expression of the most characterized lncRNA *HOTAIR* (HOX transcript antisense RNA) was significantly reduced in chondrocytes that were pretreated with lipopolysaccharide in order to suppress the inflammatory process. This transcript is able to suppress mir-138-mediated synthesis of NF-κB, since miRNA-138 is a *HOTAIR* direct target (Zhang H. et al., 2017).

Expression of the other lncRNA *ZFAS1* (zinc finger antisense 1) is increased in RA synovial tissue compared to healthy donors and can enhance migration and invasion of RASFs by directly affecting miRNA-27a (Ye et al., 2018).

lncRNA *GAPLINC* (Gastric Adenocarcinoma Predictive Long Intergenic Non-coding RNA) is overexpressed in RASFs and regulates their proliferation, migration and pro-inflammatory cytokine production by operating as a sponge for miRNA-382-5p, and miRNA-575 (Mo et al., 2018).

A possible effect of circular RNAs (circRNAs) on miRNAs in RA was revealed, too. Presumably, these transcripts complementarily bind to miRNAs thereby preventing their interaction with target genes (Zheng et al., 2017).

## Summary and Future Directions

In the last few decades, many studies have shown that epigenetic mechanisms are involved in the regulation of all biological processes in the body from impregnation to death. These functional mechanisms are involved in genome reorganization, control of gametogenesis and early embryogenesis, and play an important role in cell differentiation. Changes in DNA methylation and posttranslational modifications of histones are key epigenetic events contributing to the reorganization of chromatin into euchromatin, heterochromatin, and regions of nuclear compartmentalization, which allow to regulate gene expression by forcing them to be consistently switched on and off for the normal development of a multicellular organism. Epigenetic changes may form and appear over a long period of time, for example, during the training and organization of memory (Moosavi and Motevalizadeh Ardekani, 2016). Aberrations of epigenetic modifications can cause the development of congenital defects, hereditary diseases and multifactorial diseases including malignant tumors in different periods of life. DNA methylation, histone modifications, expression of proteins that generate or remove epigenetic marks, and ncRNAs affect inflammatory and matrix-degrading pathways and could be changed in RA. Epigenetic mechanisms play an important role in the pathogenesis of the disease.

Unlike genetic lesions, epigenetic alterations are reversible and could be modulated by diet, drugs and other environmental factors. This epigenetic flexibility suggests strategies for prevention and therapy of diseases with confirmed pathogenic role of epigenetic factors (Pashayan et al., 2016).

Therapeutic targeting of epigenetic mechanisms can be a successful approach in the treatment of chronic inflammatory diseases. Significant efforts have already been made to develop drugs that able to restore or alter the epigenetic mechanisms. DNA methyltransferase inhibitors (DNMT), 5-azacitidine (Vidaza) and 5-Aza-2′deoxy-5-azacitidine (Decitabine) are already being used to treat inflammatory conditions in pancreatitis therapy. Two types of HDAC inhibitors (HDACi) are used for treatment: pan-inhibitors with broad spectrum of action and specific inhibitors that target a specific class of HDAC enzyme. To date, the Food and Drug Administration (FDA) has approved four HDACi: Vorinostat, Romidepsin, Panobinostat, and Belinostat. These products have the minimal side effects and are mainly used for the hematological tumors treatment (Samanta et al., 2017).

Moreover epigenetic alterations are a source of diagnostic and prognostic markers. The gradual changes of epigenetic marks that may be caused by environmental factors can result in both the development and progression of pathological conditions. DNA methylation marks are the best characterized, its role is comprehensively studied in cancer development. The existence of loci and genes with differential methylation patterns, varying, respectively, the stage of the disease, was also revealed in patients during RA progression (Ai et al., 2015). However, the plasticity of the epigenome is a complication for the researches. The prognostic markers panels consisted of epigenetically modified genes could differs individually due to the variability of environmental factors affecting patients. Smoking is the most common environmental risk factor in RA development and its severity determination. In addition to directly affecting lung tissues, smoking alters the expression of sirtuins (SIRT), the proteins of the deacetylase families that are involved in modification of histone and non-histone proteins. SIRT maintain the integrity of the genome during the cellular response to stress by using epigenetic mechanisms, and are therefore key molecules in the body’s adaptation process. The change of expression of SIRT1 and SIRT6 in RASFs was demonstrated (Engler et al., 2016).

In contrast to genetic aberrations that are persist throughout life, epigenetic changes may vary in different cell populations as well as in the same cell depending on conditions and developmental stage. Since the whole blood cells, T- and B-lymphocyte populations, and SFs exhibit different DNA methylation patterns, the analysis of epigenetic markers in a mixed cell population may hamper in the correct evaluation of the epigenetic panel (Liu et al., 2013; Glossop et al., 2016).

Similarly, miRNA profiles differ across individuals, cell populations and may be affected by concomitant diseases (Huang et al., 2011). Thus, it is important to consider the extracted cell type during the development of RA epigenetic markers panel. Individual patient therapeutic response could also be predicted with the epigenetic panel use. Currently, a large number of drugs for RA treatment is undergoing preclinical and clinical trials, however, the existing data linked the epigenetic changes and response to therapy is limited. Thus, intensive investigations in this field are critically needed. International cooperation will enable the access to larger patient cohorts thereby improving the quality of studies. However, when planning international consortia, it is important to consider the differences in ethnic composition that may be reflected in epigenetic variations (Rawlings-Goss et al., 2014). Some differences that were related to race and nationality of patients were demonstrated even for genetic markers.

Nevertheless, despite of the complications, investigation of epigenetic markers is undoubtedly a great achievement of molecular biology and molecular medicine. Epigenetic changes are the earliest factors that are associated with the development of the disease before its clinical manifestation. They can be used for prevention and monitoring of patient condition and also are the earliest to reflect the effect of the drug at the cellular level, before any systemic response of organism manifested as certain symptoms. Moreover, some of epigenetic markers, such as changes in circulating miRNAs level in plasma, may be more accessible for evaluation than the other molecular markers.

## Author Contributions

MN, DZ, AL, and AZ contributed conception the review. MN, IB, DM, EK, EA, MB, and AD wrote sections of the manuscript. All authors contributed to manuscript revision, read and approved the submitted version.

## Conflict of Interest Statement

The authors declare that the research was conducted in the absence of any commercial or financial relationships that could be construed as a potential conflict of interest.
